# Dr. Ellwood Wilson

**Published:** 1889-08

**Authors:** 


### Obituary.

DR. ELLWOOD WILSON, of Philadelphia, Pa., one of the distinguished ornaments of the medical profession of the period, died on Sunday, July 14, 1889, aged 67 years.

